# A Trial of a Solar-Powered, Cooperative Sensor/Actuator, Opto-Acoustical, Virtual Road-Fence to Mitigate Roadkill in Tasmania, Australia

**DOI:** 10.3390/ani9100752

**Published:** 2019-09-30

**Authors:** Bruce Englefield, Steven G. Candy, Melissa Starling, Paul D. McGreevy

**Affiliations:** 1 School of Veterinary Science, The University of Sydney, Sydney, NSW 2006, Australia; mjstarling@fastmail.com.au (M.S.); paul.mcgreevy@sydney.edu.au (P.D.M.); 2 Scandy Statistical Modelling Pty Ltd, 70 Burwood Drive, Blackmans Bay, Tasmania 7052, Australia; burwood70@gmail.com

**Keywords:** wildlife vehicle collisions, roadkill, One Welfare, virtual fence, avoidance learning, animal welfare

## Abstract

**Simple Summary:**

The Australian state of Tasmania has a high rate of roadkill, so any method that reduces roadkill in this state deserves attention. A commercial roadkill mitigation device, which combines an auditory warning signal with flashing blue and amber lights in linked units to form a so-called virtual fence, is said to reduce roadkill by up to 90%. For the current trial, a virtual fence was installed on a 4.5-km segment of Tasmanian highway south of Hobart and roadkill was monitored on a daily basis for a period of 126 days. Sections of the virtual fence were switched on or off, according to a predetermined experimental design. Bennett’s wallabies, Tasmanian pademelons, and common brush-tail possums accounted for most of the total roadkill of 174 animals over the study period. For these three species, four complementary methods of analysis failed to reveal any significant effect of the virtual fence in reducing roadkill. This study does not confirm previously reported estimates of reduction in roadkill rates of 50%–90%.

**Abstract:**

When wildlife and motor vehicles collide, the result for the animals is often death (roadkill). A commercial roadkill mitigation device that forms a so-called virtual fence (VF), is said to reduce roadkill by up to 90%. A field trial to test its effectiveness was undertaken along a 4.5-km segment of a Tasmanian highway subdivided into 6 equal sections. A total of 126 days of monitoring of roadkill by species was conducted, with alternate sections being switched on or off, according to a variation of Crossover and Multiple Before-After-Control-Impact experimental designs that divided monitoring into five periods. From the six sections over the five periods, the 30 aggregated values of daily counts of roadkill for each species were modelled. Bennett’s wallabies (BW) *(Notamacropus rufogriseus)*, Tasmanian pademelons (TP) *(Thylogale billardierii)* and common brush-tail possums (BP) *(Trichosurus vulpecula)* accounted for most of the total roadkill of 174 animals. Although initially there appeared to be an effect, linear model fits to standardised roadkill rates were not statistically significant for each of BW, TP, and BP using each of the Crossover, Multiple Before-After-Control-Impact, and simple On versus Off comparisons. Adjustment for spatial and temporal trends using a Generalised Additive Model with Poisson error also failed to detect a significant VF effect. A simulation study used to estimate the power to detect a statistically significant reduction in roadkill rate gave, for median estimates of reduction of 21%, 48%, and 57%, estimates of power of 0.24, 0.78, and 0.91, respectively. Therefore, this study failed to confirm previously reported estimates of reduction in roadkill rates claimed for this VF of 50%–90%, despite having adequate power to do so. However, point estimates obtained for these three species of reductions ranging from 13% to 32% leave open the question of there being a real but modest effect that was below statistical detection limits.

## 1. Introduction

Road infrastructure is expanding rapidly on a global scale as industrialisation and urbanisation increase [1,2]. One consequence is a global rise in animals being killed or injured (roadkill) in wildlife vehicle collisions (WVC) in Europe [3], the Americas [4] and Australia [5], and a rising number of injured and orphaned animals being rescued by welfare organisations [6,7,8,9]. WVC can have serious consequences for animals and humans, i.e. death or injury, and can affect the environment through species decline [10,11,12,13,14]. The ‘One Welfare’ concept [15,16], where animal health, human well-being, socio-economic development and environmental sustainability are inexorably linked, might suggest that any reduction in WVC could have strategic value, such as fewer car insurance claims and less roadkill for tourists to encounter. In Australia, a 10% reduction in WVC would mean up to 40,000 fewer mammalian roadkill victims each year [5].

Three main approaches to mitigating the problem of WVC, which can be undertaken individually or in combination, are infrastructure management, changing human behaviour and changing animal behaviour. Infrastructure management includes building roadside fences, culverts and land-bridges, but these are costly, ranging from AUD 50,000 for a culvert to AUD 2 million for a land-bridge [17,18]. Changing human behaviour focuses chiefly on attempts to change the behaviour of drivers but this has proven to be elusive [12,19,20], although studies are starting to merge the biological and social sciences in a bid to address this [21,22]. Changing animal behaviour to reduce WVC may involve the use of devices that trigger a flight response and alert wildlife to approaching traffic. However, when subjected to scientific evaluation, few of these methods have been shown to be effective and have little value [23,24,25,26,27]. 

Since the studies that have attempted to change animal behaviour in the proximity of roads have met with limited success, there is a need for further exploration in this area. The recent development of lithium-polymer rechargeable batteries and thin film solar-cells, combined with very low power consumption, has enabled the production of active roadside systems. These produce auditory and optical signals to alert animals to approaching vehicles, which are intended to trigger a response from animals of avoiding or departure from the road. One such device is a solar-powered, transport system sensor/actuator manufactured in Austria (iPTE Traffic Solutions Ltd. 8054 Graz/Austria Mantscha-Wald-Weg 48, Austria). The units are designed to produce a virtual fence (VF) along the roadway and to work from dusk to dawn, alerting crepuscular and nocturnal animals. An internal light sensor in the unit detects an approaching vehicle’s headlight at a standard threshold of 150 lux. This causes an optical/acoustical alert system to be triggered. According to the manufacturer, *‘The acoustic sound of the warning sequence raises the attention of the animals and the flashing lights makes the animals feel uncomfortable and leave the road area’.* A five-year trial of these devices in Austria concluded that there was a 90% sustainable reduction of roadkill resulting from WVC [28,29]. Subsequently, a three-year trial in Tasmania, Australia, concluded that roadkill was reduced by over 50% and that *‘these devices have enormous potential to substantially reduce roadkill rates’* [30]. However, at other test sites in other countries, these results could not be reproduced. For example, in the UK, USA and Hungary, the devices did not have any significant effect in reducing roadkill from WVC [28]. The aim of this study was to assess the operation of the VF and to explain the divergent findings on its effectiveness. The experimental design employed true spatial and temporal replication.

## 2. Materials and Methods 

### 2.1. Study Site and Data Collection

In April 2018, the Tasmanian Department of State Growth, Infrastructure, Energy and Resources erected a VF system (Virtual fencing unit DD430-B Gen_3) produced by IPTE Traffic Solutions Ltd, (8054 Graz, Austria, Mantscha-Wald-Weg 48) along a 4.5-km section of the Huon Highway, a single carriageway road with three lanes, from Lesley Vale (42^0^ 58’ 0.49S 147^0^ 14’ 51.57E) to Sandfly (42^0^ 58’49.18S 147^0^ 12’26.53E). (See Supplementary Material for operating characteristics of the VF system including Figure S1 showing sound levels for general traffic, the VF unit, and background noise).

The 4.5-km section of highway [31] is mostly straight with some sweeping bends. Proceeding north-east to south-west, the highway has a gentle decline but becomes steeper over approximately the last 1.5 km. Rough pasture abuts the highway, with intermittent copses of eucalypts and light undergrowth either side of the clear gravel and grassy verges, ranging in width from approximately 5 to 15 m. The vegetation becomes more dense woodland alongside the steeper south-western sections. A traffic counter (Vehicle Classifier System, MetroCount, 15 O’Connor Close, North Coogee, W.A. 6163 Australia) was located 910 m west of the Huon HWY-Leslie Road intersection. The counter accurately approximates the number of vehicles using the full extent of the trial site, their speed, and time of their passage on a continuous basis. The operational status of the VF units was checked on a weekly basis. 

The VF was divided into six, equal in length, segments, using Google Maps to measure and allocate exact GPS co-ordinates of the start and end of segments [31]. A buffer section of 750 m at each end of the virtual fence resulted in eight segments being monitored. The eight segments were searched for roadkill on a daily basis over 18 weeks. Each roadkill was photographed with a time, date, and GPS stamp and then left in place (see Supplementary Material). Removing carcasses would have reduced the likelihood of scavengers such as Tasmanian devils (*Sarcophilus* *harrisii*) and quolls (*Dasyurus maculatus* and *Dasyurus viverrinus*) becoming roadkill [32] (also see Figure S2, Supplementary Material).

### 2.2. Experimental Design Including Treatment Allocation (VF Off vs On) and Data Aggregation/Standardisation

The experimental design, drawing data from only the six sections (i.e., Sections 2 to 7) that had the VF installed, was a combination of a replicated BACI [i.e., Multiple Before-After-Control-Impact (MBACI)] design [33], and a Crossover design. Crossover designs are commonly used in medical research [34,35] and, in our application, the VF switched Off and On are analogous to a placebo and a medical intervention, such as administering a drug, respectively. The Crossover design involved dividing the six sections (contiguous Sections 2 to 7, proceeding westward) into two blocks, with Block 1 composed of Sections 2, 4 and 6, and Block 2 composed of Sections 3, 5 and 7 (Table 1).

#### 2.2.1. Monitoring Periods

A period of 42 days of monitoring of roadkill by species, starting 26 March 2018, was conducted prior to the VF being switched on, being split into a pre-trial monitoring period of 14 days, starting from the 26 March, then a 28-day period from when the VF was installed (on 1 May) but not switched on. For the next 28 days, the three sections in Block 1 had their VF switched on. The road was then monitored for a 14-day period with the VF switched off on all sections. In the following 28-day period, only the three sections in Block 2 had their VF switched on. Monitoring proceeded for a subsequent and final 14-day period with the VF switched off for all sections. Total days of monitoring were 128, half of which were with the VF switched on (Table 1).

#### 2.2.2. Aggregation and Standardisation of Roadkill Rates

Counts were aggregated across the Pre-trial (14 days) period and the subsequent Pre_All_Off (28 days) period within each section into a single count for the statistical analyses. Period 2 in the statistical analyses refers to this combined period of 42 days. The Period factor, therefore, takes the five labels of Period 2 to Period 6. From the six sections over the five periods, 30 aggregated values of daily counts of roadkill were obtained separately for each species and used for formal statistical analyses. The roadkill counts were analysed as raw counts or standardised rates depending on the statistical model that was fitted (see Supplementary Material). 

### 2.3. Statistical Analyses

We present four different analyses because they each exploit different aspects of the experimental design and, in doing so, they either use different subsets of the data or, in the case of the omnibus LM test and the GAM, the same full dataset. The latter attempts to improve the precision of estimates of reduction in roadkill rates due to the VF by adjusting for any spatial or temporal smooth trends in roadkill rates. The Crossover analysis compares On vs Off across different spatial units for a fixed temporal unit within Block, while the MBACI analysis contrasts Before and After (i.e., Off vs On, respectively) within, effectively fixed spatial units (i.e. this contrast removes spatial effects).

#### 2.3.1. Crossover, MBACI, and Omnibus Off vs. On Analyses

For these analyses the response variable used was standardised roadkill rate using either period length (days) or traffic count standardisations. The Crossover analysis compares roadkill rates in Block 1 between On (Period 3) and Off (Period 5) and in Block 2 for Period 5 and Period 3 with this order of periods reflecting VF On vs VF Off. Means were graphed by block and visually overlaid. If there was a substantial reduction in mean standardised roadkill rate due to the virtual fence operating, then the lines connecting means within blocks should intersect (i.e., when graphed with order on the abscissa scale of Period 3 then Period 5 the slope should be positive for Block 1 and negative for Block 2). A formal test of the significance of the effect of the VF used a linear mixed model (LMM) [36] with period factor (two levels) as a main effect, a single degree of freedom contrast between Off and On, and a random section effect for the data restricted to just these two periods. Carry-over effects [34] were assumed to be negligible due to the 14-day period with the VF switched off on all sections. The *lmer* function from the R-software [37] library *lme4* was used to fit the LMM. Note that this crossover test can also be considered a contemporaneous comparison since it equivalently tests the difference between On versus Off within each period.

The MBACI design consisted of pairing adjacent On/Off sections in that order: For Period 3, these were Sections 2/3, 4/5, 6/7, while for Period 5, these were reversed as 3/2, 5/4, and 7/6. So, in each case the On sections within a block are considered the Impact (Treatment) site, and the Off sections the Control site. Further, for Block 1, Period 2 represents the Before versus Period 3 as the After in BACI notation, so that subtracting the mean rate for the former from that of the latter gives the contrast estimating the effect of the VF. Similarly, for Block 2, Period 4 represents the Before versus Period 5 as the After comparison. Therefore, there were six spatial replicates of the BACI design; three in each block. Similar to the crossover, the means, and their standard error (SE) bars are graphed separately for each block. However, for the MBACI components, there were four means and two connecting lines for each block with means within each block graphed, with abscissa being the factor levels in the order of Before and On (denoted After for a MBACI design where On or After for the Control section/periods simply means that they were observed contemporaneously with the adjacent VF On section within the pairs). If there was a substantial reduction in mean standardised roadkill due to the virtual fence operating then there should be a substantial decline in mean from Before to On for the Impact section/periods, consistently, across the two blocks, and a similar decline should not be seen for the Control section/periods. For the Control sections, a minor decline, no change, or an increase might occur in mean standardised roadkill. To take into account any change within the Control section/periods contemporaneously with the Impact section/periods, this change was subtracted from the mean change for the Impact section/periods and the corresponding estimate of the standard error of this adjusted mean contrast was obtained. 

A final general or omnibus test was conducted for all periods, using a simple On versus Off factor (denoted as VF_On_vs_Off) fitted in a linear model (i.e., with no Block main effect or interaction with VF_On_vs_Off included). The percentage change (i.e., 100 times the Off minus On mean divided by the Off mean) was calculated and the standard error of this percentage approximated by a second order Taylor series approximation [38] and these values are given in the Results.

#### 2.3.2. Generalized Additive Models

To account for differing length of time periods, length of sections, or traffic count for the period, the raw counts were used as the response variable in a Generalized Additive Model (GAM) [39] with Poisson error, Naperian log link function, and an offset of the sum of the log of the number of days in the period (or alternatively the log of the traffic count for the period) and the log of the length of the section. The GAM allows trends to be modelled as empirical smooths (such as a cubic smoothing spline). GAMs were used to adjust estimates of the effect of the VF by effectively removing the effect of smooth trends in each of the Section sequence number (i.e., a very close approximation to using the distance to the spatial locations of section midpoints from the start of Section 2) and the period midpoints (i.e., where for Period 2 this was taken as the days from March 25 to May 1), using cubic smoothing splines with basis dimension of three for both splines. These splines were added to the linear predictor of the GAM and combined with the general On versus Off contrast (i.e., effect). The percentage reduction in standardised roadkill was obtained directly from the GAM as 100*{1-exp(par_est)} where par_est is the parameter estimate for the contrast in question with corresponding standard error approximated using a first-order Taylor series approximation by 100*exp(par_est)*SE(par_est) where SE(par_est) is the estimated standard error. The GAM was fitted using the *gam* function from the R *mgcv* library [39]. Comparison of the fit of the GAM that used the component of the offset of log of Period length compared to that using the log of traffic count was made using the Akaike Information Criterion (AIC) [40]. The Dunn-Smyth type quantile residuals [41] were plotted against the standard normal quantiles to assess whether the assumption of Poisson distributed counts was reasonable using functions from the R *statmod* library.

#### 2.3.3. Simulation Study to Estimate Power to Detect a Statistically Significant Reduction in Roadkill

To deliver true replication of On and Off treatments (i.e., section by period combinations), the study design focused on providing a valid comparison, both spatially and temporally. Accordingly, there was less replication of the On treatment (six replicates) than the Off treatment (24 replicates). To investigate the power to detect a statistically significant reduction in roadkill using this study design, using an assumed Poisson distribution for counts and the spatial and temporal trends estimated by the GAM, the fitted GAM for the BW data was used as a baseline operating model. 

To investigate a range of percentage roadkill reductions, including the estimate from the fit to the actual data, the On and Off predicted means for section by period combinations obtained from the GAM were jointly deflated and inflated, respectively, each by 0%, 10%, 20%, and 30%. For each of these four sets of predicted values, a random Gaussian estimation error from the fit of the GAM was added to the linear predictor scale. A random Poisson value for each section by period combination was drawn based on these scaled and perturbated linear predictor values to generate 1000 sets of these 30 random values for each deflation/inflation percentage. The GAM was refitted to the 1000 datasets and the percentage reduction estimated for each deflation/inflation percentage. Using these 1000 estimates of percentage reduction, the median estimate was determined and used as the assumed alternative hypothesis value to test (i.e., where the null hypothesis is a zero value for percentage reduction). The critical value for the null hypothesis test was taken to be the upper 95% quantile of the 1000 sample estimates minus the median estimate (i.e., a one-sided test). The power of the test (i.e., probability of rejecting the null hypothesis when the true value of percentage reduction is as estimated by the median value corresponding to one minus the probability of a Type II error) was calculated as the proportion of the 1000 simulations where the estimated percentage reduction was greater than the critical value. Thus, the power was estimated for each median estimate of percentage reduction corresponding to each deflation/inflation percentage, since these last percentages were more straightforward to use as inputs than trying to manipulate percentage reduction directly.

The above analysis of power considers only one study site (i.e., the site of this study). So, the power analysis was extended to consider a random sample of *S* sites with the same study design. To do this, transformations of the percentage reduction that gave a constant standard error across the range of estimated percentage reductions in the above simulation study were examined empirically. Once this transformed estimate was obtained, the corresponding average standard error was divided by the square root of *S* to give an estimate of the standard error of the mean transformed reduction estimated across *S* replicates of the design. Classic power analysis (Steele and Torrie, 1960) was carried out using a t-distribution for the transformed estimate, a target value for percentage reduction, and the value of *S* in order to obtain an estimate of the power to detect the target reduction in roadkill rate due to the VF. Note that the power will be under-estimated to a degree dependent on the magnitude of between-site variance in the site-specific estimates of percentage reduction since, in the absence of any reliable estimate of its magnitude, this variance was assumed to be negligible in the extended power analysis.

## 3. Results

### 3.1. Vehicle and Roadkill Data

Daily total traffic count (i.e., across axle types and speed classes) for the dusk to dawn period separately for westbound and eastbound traffic on the 6 km stretch of road studied are shown in Figure S3 (Supplementary Material). Average (± one standard deviation) and maximum daily traffic speed between dusk and dawn for the study period are shown in Figure S4 (Supplementary Material). Over the period 25 March to 20 August, 388,595 vehicles were recorded between dusk and dawn (combining eastward and westward traffic) with 25.4% recording a speed above the limit of 100 km.h^−1^ and 3.5% recording a speed in excess of 120 km.h^−1^.

Bennett’s wallabies (BW) *(Notamacropus rufogriseus)*, Tasmanian pademelons (TP) *(Thylogale billardierii)* and common brush-tail possums (BP) *(Trichosurus vulpecula)* accounted for most of the total roadkill of 174 animals. The total roadkill disaggregated by species and period is shown in Table S1 (Supplementary Material). Spatial locations for BW, TP, and BP found dead are given by Figure S5 (Supplementary Material) and show an even spread along the trial 6 km road section for each of these three species. Table 2 gives estimates of standardised roadkill rates by the status of the fence as Off versus On for the three most prevalent (see Table S1) roadkill species. Rates were calculated using counts for trial sections 2 to 7. Raw rates were obtained by standardisation after aggregating raw counts across these sections and periods within Off versus On treatment status. 

### 3.2. LM, LMM, and GAM Outputs

The mean rates of roadkill for both standardisations obtained from the LM estimates for the general VF_On_vs_Off factor are given in Table 2, along with corresponding estimates of the percentage reduction in roadkill rate due to the VF being switched on. Note that the raw mean rates are different from the LM-estimated rates since the raw rates are effectively weighted versions of the modelled rates with weights corresponding to the standardisation variable for each period and section. Similarly, the GAM estimates of the percentage reduction in roadkill rate are given after adjusting for the smooth trends (described in 2.3.2) estimated across periods and sections. These smooth trends are shown for the “month^−1^ km^−1^” standardisation (i.e., corresponding to the appropriate offset term in the GAM) for BW, for smooths in section number and period midpoint in Figures S6 and S7 (Supplementary Material), respectively. 

The percentage reduction estimate, its estimate of standard error, and the formal test of significance obtained from the GAM for VF_On_vs_Off are shown in Table 2. The VF_On_vs_Off parameter estimate (par_est) for the default “set to zero” parameterisation (i.e., giving an On minus Off estimate) was not statistically different from zero (*p* > 0.1) for each of the three main species for both the LM and GAM fits. Dunn and Smyth quantile residuals from the GAM fit indicated that the Poisson assumption was reasonable for each of the three species modelled. The results for the GAM, using the log of traffic count as offset, were very close to those presented above and gave a difference in AIC statistic between these two models that was less than 1% across all three species. Therefore, either model can be used for inference since they have very close to the same goodness of fit to the data. The percentage reduction in rate, using the general On versus Off contrast (Table 2), was generally higher (by between 7% and 12%) when using the traffic count-based standardisation but the standard errors were also in general higher. Using either standardisation, the GAM parameter estimate (par_est) was not significantly different from zero (*p* > 0.1). We concentrated on the results using the log of period length as offset (i.e., effectively the month-based standardisation) as it was easier to interpret (i.e., in the absence of traffic counts). 

**Figure 1:**
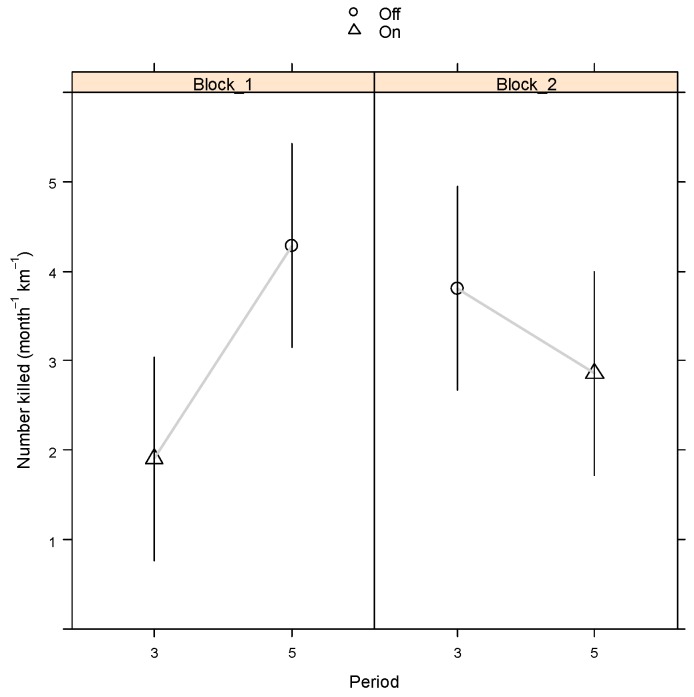
Standardised roadkill (month^−1^km^−1^) for Bennett’s wallaby for Periods 3 and 5 for Crossover means by Block. Single Standard Error (SE) bars are shown.

**Figure 2:**
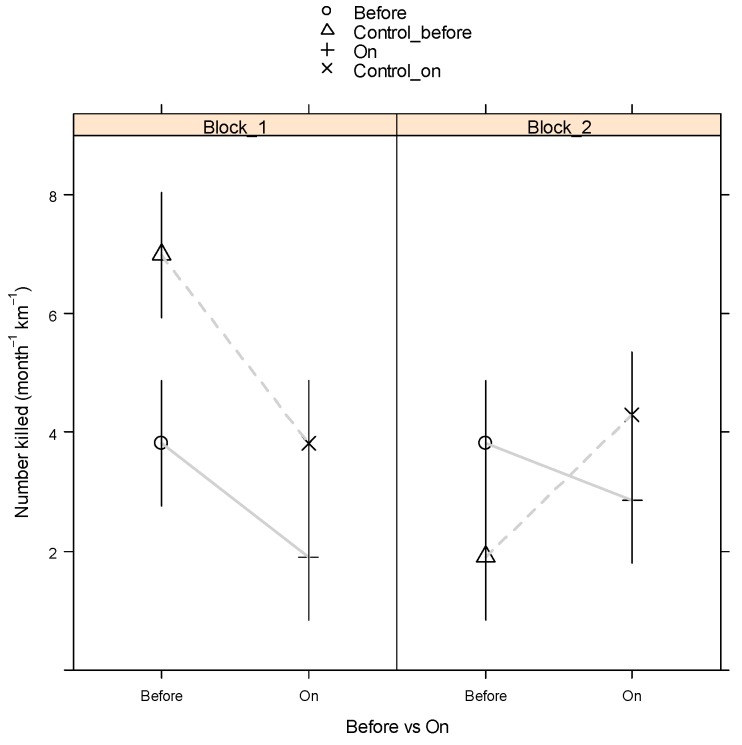
Standardised roadkill rate (month^−1^km^−1^) for Bennett’s wallaby for Periods 2 to 5 for MBACI means by Block. Single SE bars are shown.

Table 3 gives the contrast means (using the month-based standardisation) corresponding to On minus Off mean for the crossover, MBACI and general VF_On_vs_Off analyses by Block. For the MBACI contrast of Impact: Before versus After, the adjustment for the MBACI Control contrast is also given in Table 3. For the crossover, the combined (across blocks) means and standard errors were obtained from the LMM. As seen from Table 3, none of the contrasts that quantify the effect of the VF were statistically significantly different from zero (p > 0.1). Figure 1 and Figure 2, respectively, are presented for BW to demonstrate the crossover and MBACI analyses graphically for species observed. Similar graphs could be constructed for TP and BP from Table 3 (which have not been shown for brevity).

The fitted GAM with effective standardisations of either period length in days or as dusk-to-dawn traffic counts, showed no significant reduction in roadkill rate (Table 3) (*p* > 0.1) for the VF. Point estimates obtained from the fitted GAM indicate a moderate reduction of between 23% and 32% for BW and TP, respectively, albeit with large uncertainty bounds as reflected in the large relative standard errors (Table 2). For Bennett’s wallaby, there was an indication of a reduction in roadkill rate (month^−1^km^−1^) due to the VF (Figure 1). Both Blocks 1 and 2 had an apparent drop in mean roadkill rate when the VF was switched on, so that overlaying the trends for the two blocks revealed a cross-over effect that confirmed point-estimates of a reduction in rate. However, these reductions were not significant (*p* > 0.1) either when considering blocks separately or when averaging across blocks (Table 3). The MBACI analyses showed (Figure 2) that, for Block 1, the decrease in rate from Before to After the VF was switched on for the Impact sections (2, 4 and 6) was exceeded by a greater decrease for the Control sections (3, 5 and 7), which infers there was no reduction due to the VF switched on relative to being switched off. For Block 2, a relatively minor decrease in rate for the Impact sections can be inferred to be an underestimate due to the increase in rate for the Control sections. However, averaging across blocks, the overall reduction was only minor. None of the above effects were statistically significant, with the exception of the reduction for the MBACI analysis and the Control sections for Block 1 (*p* < 0.05, Table 3), where this last effect clearly is not the result of the VF being switched on.

### 3.3. Simulation Study of Power

Table 4 gives the results of the simulation study investigating the power of the study design and the GAM fitted to the BW data to detect a statistically significant reduction in roadkill rate of Bennet’s wallaby.

For the extended power analysis, the transformation log(1-%reduction/100) gave standard error of estimates, corresponding to median percentage reductions (Table 4), ranging from 0.43 to 0.48 with a mean of 0.45. The untransformed median percentage reductions (Table 4) and alternative transformations of log(%reduction/100) gave much larger ranges in standard error estimates. Using the above mean of 0.45 for the standard error and the estimated residual degrees of freedom from the fit of the GAM of 25.7 giving the required t-distribution with degrees of freedom 25.7 × *S*, a target of a 25% reduction, a value of *S* of 16 (i.e., our study replicated at each of 15 other sites), the power to detect such a reduction was 0.82. For a target of 10% reduction and *S* of 100, the power was 0.76.

## 4. Discussion

### 4.1. Efficacy of Virtual Fencing

Mitigating the effects of wildlife vehicle collisions, and subsequent roadkill, is a world-wide goal for road managers. Many different measures have been studied and trialled to determine their effectiveness [2,42]. However, only two studies, Schalk et al. [28] and Fox et al. [30], have reported on the efficacy of a virtual fence of the design tested in the current study. The rigour of the science of the latter has been questioned, with Coulson and Bender [43] stating that ‘there are a total of eight methodological flaws ranging from imprecise measurements, confounding effects of treatments, low statistical power, violation of test assumptions and failure to consider habituation’. The design of the current study employed true spatial replication as well as temporal replication (i.e., in contrast to the lack of spatial replication in Fox et al. [30]) of On and Off treatments (i.e., section by period combinations) and therefore provided a valid comparison both spatially and temporally. To confirm the power of the study to detect a statistically significant reduction in roadkill using this study design, the simulation study showed that for true reductions of 21%, 48%, and 57% the power (i.e., 1- probability of a Type II error, often denoted as a false negative) was estimated at 0.24, 0.78, and 0.91, respectively. Thus, a marginal reduction of around 20% (i.e., of similar magnitude to the point estimates obtained for BW and TP in the current study) had low power to reveal such a reduction as statistically significant but, for the reported reduction of 50% for TP reported by Fox et al. [30] (and with reference to European estimates in the order of 80%–90%), it had high levels of power. 

A limitation of our study is the relatively short monitoring period of 18 weeks compared to that of Fox et al. [30]. The much higher roadkill rate in our study compared to that of Fox et al. [30] compensates to a considerable degree for the shorter monitoring period since the relative standard deviation for a Poisson count variable decreases as the mean rate increases, thus improving the power to detect differences in mean rates. However, the current study, on its own, was reliably estimated to have sufficient statistical power to confirm estimates of a substantial (i.e., >50%) reduction in roadkill rates due to operation of the VF, but failed to do so. 

The main research question investigated by the statistical modelling and hypothesis testing in the current study was whether the VF reduced the roadkill rate for any of the species that had sufficient numbers of roadkill within the study period to give reliable estimates of average roadkill rates. A linear model for standardised rates of roadkill using either days combined with section length, or dawn-to-dusk traffic count combined with section length, did not indicate the VF would significantly reduce the rate of roadkill for any of the three major roadkill species (*p* > 0.1). These tests were obtained from three separate analyses using crossover, MBACI, and general VF On versus Off comparisons. The fitted GAM with effective standardisations of either period length in days or as dusk-to-dawn traffic counts, showed no significant reduction in roadkill rate for the VF. 

These four analyses are complementary and none of the tests they imposed could detect any significant effect of the VF on roadkill rates. Combined with the simulation study that estimated the power to detect effects of a range of magnitudes, the current study indicates that if the true effect size corresponded to any of the point estimates (i.e., an approximate 20% to 30% reduction) obtained from the different analyses, it was below that detectable using the study design and dataset obtained. There was a reasonably uniform distribution of roadkill along the entire section of highway studied (Figure S5). Any "spill-over" effects are likely to have been minor and would not have affected our estimation of the effect of the VF to any practical degree. Had there been any significant “spill-over”, combined with a substantial reduction in roadkill due to the operation of the VF, then roadkill in the start or end of the sections with VF turned off would have been lower than expected due to operation of the VF in the adjacent "On" section. However, this is not apparent from Figure S5.

A notable feature of the results from this study is the high rate of roadkill for BW and TP, by a factor of nine and five times, respectively, compared with the rate for the unfenced road section reported by Fox et al. [30] of 0.347 month^−1^km^−1^ for BW and at 0.627 month^−1^km^−1^ for TP. However, while common brush-tailed possums (BP) were a major component in the current study, with a rate of 1.39 month^−1^km^−1^, they were a minor component in the Fox et al. study, with a rate of 0.026 month^−1^km^−1^. The much higher roadkill rates for Bennett’s wallaby (BW) and Tasmanian pademelon (TP) in the current study could possibly be attributed to much higher dusk-to-dawn daily traffic counts, although no traffic count data were reported in the Fox et al. study. BW, TP, and BP are crepuscular/nocturnal feeders, so most roadkill occurs between dusk and dawn [19]. There is also the possibility that the two habitats contained differing animal population densities. The Arthur Highway on the west coast of Tasmania runs through coastal scrubland whereas the Huon Highway of South East Tasmania runs through farmed grazing land combined with native bushland. Larger population sizes due to the greater availability of nearby pastureland and native bushland for grazing would be expected at the Huon Highway site [44].

### 4.2. Limitations of the Virtual Fence and Future Research

The secondary purpose of the trial was to explore why the VF may be ineffective at this location. In correspondence with the current research team, the manufacturers state: *‘effectiveness* [of the devices] *is speed dependent’*. That speed can be of significance with roadkill as demonstrated by Hobday and Mistrell [19] although they measured driver reaction times rather than animal reaction times. Night-time driving speeds needed to be below 80 km/h^−1^ if roadkill rates were to be reduced. Average driving speeds recorded during the VF trial were well above the 80 km/h^−1^ suggested by Hobday and Mistrell, with the highest recorded speed being 189 km/h^−1^. At 189 km/h^−1^ and with the VF being triggered by headlights from 150 m, the latency for the vehicle to reach an animal at the VF site would be 2.85 s, whereas at 140 km/h^−1^ it would be 3.85 s. Both delays are likely to be insufficient for an animal to react and leave the road, so we estimate that the VF would be ineffective in these circumstances. This is confirmed in the VF manufacturer’s literature. In the section ‘Constraints on the vehicle speed’, they give the time to react and then leave the road as 6 s with the sensor-range at 150 m on low beam and maximum vehicle speed of 90 km/h^−1^ [28,29]. At the speed recommended by Hobday and Mistrell (80 km/h^−1^) it would take 6.8 s for a vehicle to reach an animal, sufficient time for the animal to react and leave the road. However, for the VF to be effective, it must produce a response from the wildlife and it is unclear whether the sound and light stimuli of the VF produce such a reaction. No research has been undertaken in Tasmania to study the reaction of native animals to stimuli such as the sound and light emissions of the VF. This would be a useful addition to understanding possible limitations of the VF. Further possible limitations are described in the supplementary material.

Further, well-designed field trials with valid spatial and temporal replication and sufficient numbers of roadkill are required to obtain an acceptable relative precision of the estimate of reduction in rate, particularly if the anticipated reduction is substantially less than 50%. The extended power analysis indicates that our study would need to be replicated at an additional 15 sites to be able to detect a 25% reduction with adequate power. To detect a 10% reduction, an additional 99 sites would be required. Note that this number of sites is conservative since we were unable to take into account between-site variance in estimates of percent reduction in the extended power analyses. Before conducting any extensive program of field trials to more precisely estimate the degree of mitigation corresponding to reductions of the order of 20% or less, this needs to be balanced against the cost of such a program as well as the cost/benefit of such moderate reductions compared to alternative roadkill mitigation strategies. In addition, there are concerns about the theory of how the VF operates (see Supplementary Material). We recommend that investigation of potential improvements in the effectiveness of the VF be conducted before more research trials or operational implementation of the VF are undertaken.

## 5. Conclusions 

In contrast to comparable studies of this virtual fence (Fox et al. [30]), the current study site and observation period, combined with the experimental design, strict measurement protocols, and data analysis methods used did not reveal a statistically significant reduction of the order of 50% or greater even though there was adequate power to do so. For the three dominant roadkill species of BW, TP, and BP, all four complementary methods of analysis failed to find any statistically significant positive effect of the virtual fence in reducing roadkill.

The data and R-code used in this study can be provided on request from the senior author.

## Figures and Tables

**Table 1:** Periods with On-Off Periods 3 and 5 disaggregated to On and Off Blocks excluding buffer sections 1 and 8 (Block 1: Sections 2, 4 and 6; Block 2: Sections 3, 5 and 7). The periods when the virtual fence (VF) was switched on are italicised.

Period Label	Pre-trial	Pre_All_Off	Block1_On	Post1_All_Off	Block2_On	Post2_All_Off
Period No	1	2	*3*	4	*5*	6
Start Date 2018	26/03	1/05	*28/05*	25/06	*9/07*	6/08
End Date 2018	8/04	28/05	*25/06*	9/07	*6/08*	20/08
Period (days)	14	28	*28*	14	*28*	14

**Table 2:** Roadkill rates for three most prevalent roadkill species. Number killed for VF “On” versus “Off” and standardised rates using a general VF “On” vs “Off” factor/contrast estimated using all periods and Sections 2 to 7. Raw standardised rates and resultant percentage reduction based on total counts appear in the second, third, and fourth columns followed by model-based standardised rates and resultant percentage reductions with corresponding standard errors. For raw rates, the standardisation used total days of VF “On” apportioned by the fraction of sections switched on in Periods 3 and 5 and similarly for VF “Off”. Vehicle counts were similarly apportioned.

Species	Total Counts			Rate (number.month^−1^km^−1^)			Rate (number.100kVeh^−1^km^−1^)		
	VF Off	VF On	Total	VF Off	VF On	%Reduction	VF Off	VF On	%Reduction
Wallaby (BW)	58	10	68	3.946	2.381	39.66	4.986	2.612	47.60
Pademelon (TP)	48	10	58	3.265	2.381	27.08	4.126	2.612	36.69
Possum (BP)	23	5	28	1.565	1.190	23.91	1.977	1.306	33.93
				Rates from LM estimates (SE) for general VF_On_vs_Off factor					
Wallaby (BW)				3.194 (0.490)	2.381 (0.980)	25.5 (39.3)	3.951 (0.649)	25.5 (39.3)	33.1 (41.7)
Pademelon (TP)				3.492 (0.411)	2.381 (0.823)	31.8 (29.9)	4.614 (0.595)	31.8 (29.9)	42.2 (32.4)
Possum (BP)				1.389 (0.367)	1.190 (0.734)	14.3 (68.6)	1.818 (0.483)	14.3 (68.6)	27.3 (67.9)
				Rates from GAM estimates (SE) for general VF_On_vs_Off factor					
Wallaby (BW)						23.0 (27.7)			31.3 (24.7)
Pademelon (TP)						32.2 (23.9)			29.4 (26.0)
Possum (BP)						12.5 (45.5)			21.5 (40.8)

**Table 3:** Roadkill rates for three most prevalent roadkill species. Linear model contrast parameter estimates for standardised roadkill rates (number.month^−1^km^−1^).

	Crossover Contrast ^a^ (Periods 3 and 5)					
	Block 1		Block 2		LMM/LM Estimate	
Species	Estimate	SE	Estimate	SE	Estimate	SE
Wallaby (BW)	−2.381	1.615	−0.952	1.615	−1.667	1.080
Pademelon (TP)	−1.429	1.782	0.476	1.782	−0.476	1.230
Possum (BP)	0.476	1.166	−1.905	1.166	−0.714	0.673
	MBACI contrast 1 ^b^ (Impact: Before vs After) (all periods except 6)					
Wallaby (BW)	−1.905	1.495	−0.952	1.495	−1.429 (−1.032)	1.057 (1.495)
Pademelon (TP)	−0.794	1.611	0.476	1.611	−0.159 (0.794)	1.139 (1.611)
Possum (BP)	0.635	1.365	0.476	1.365	0.556 (0.238)	0.966 (1.365)
	MBACI contrast 2 ^c^ (Control: Before vs After) (all periods except 6)					
Wallaby (BW)	−3.175 **	1.495	2.381	1.495	−0.397	1.057
Pademelon (TP)	−0.952	1.611	−0.952	1.611	−0.952	1.139
Possum (BP)	0.159	1.365	0.317	1.365	0.317	0.966
	On vs Off contrast ^a^ (all periods)					
Wallaby (BW)	−0.833	1.575	−0.794	1.575	−0.813	1.095
Pademelon (TP)	−1.508	1.271	−0.714	1.271	−1.111	0.920
Possum (BP)	0.516	1.047	−0.913	1.047	−0.198	0.821

^a^ A negative estimate indicates a reduction in standardised roadkill when the fence was switched on. ^b^ A negative estimate indicates a reduction in standardised roadkill when the fence was switched on. Values within brackets are the Impact values after subtracting the corresponding Control estimates. ^c^ A negative estimate indicates a reduction in standardised roadkill when the fence for the control comparison for the sections of the block switched off both before and during the periods corresponding to the On block. If no probability level is given then *p* > 0.1. ** Probability level 0.025–0.05.

**Table 4:** Power calculation using 1000 simulations for each artificial inflation/deflation level of the VF Off/On Bennett’s wallaby counts. Simulations used Poisson random draws for each of the 30 combinations of Period by Section using Poisson means calculated as predicted means from the GAM fitted to the original data with an additional Gaussian random prediction error added on the linear predictor scale.

“On” Deflation “Off” Inflation (%) of Prediction	Median ^a^ Percentage Reduction (%)	Standard ^b^ Deviation (%)	Median Standard ^c^ Error (%)	Power ^d^ to Detect Median Reduction (P-level)
0	20.96	34.32	27.87	0.242
10	36.69	28.75	23.25	0.543
20	48.08	22.60	19.69	0.783
30	57.32	20.38	16.94	0.907

^a^ Median of 1000 simulations and GAM estimate of VF_On_vs_Off factor On effect (Par_est), with percentage reduction due to the VF switched on, given by 100*{1-exp(Par_est)}. The GAM also included cubic smoothing splines as fitted to the original data. ^b^ Standard deviation of the 1000 simulation estimates of percentage reduction. ^c^ Median of 1000 simulation estimates of the standard error of the percentage reduction given by 100*exp(Par_est)*SE_Par_est, where SE_Par_est is the standard error of the estimate (Par_est). ^d^ 1-Probability of a Type II error, where a Type II error is accepting the null hypothesis that the percentage reduction is zero when it is false as given by the alternative hypothesis that the true percentage reduction is given by the median percentage reduction. Note that the critical value of the null hypothesis test was the difference between the 95% quantile of the 1000 estimates of percentage reduction minus the median of these 1000 estimates.

## Supplementary Materials

The following are available online at https://www.mdpi.com/2076-2615/9/10/752/s1, Figure S1: Measurement of sound volume for general traffic, general background, and virtual fence, Figure S2: Possum feeding at the base of a VF post, Figure S3: Daily traffic counts between dusk and dawn, Figure S4: Average and maximum daily traffic speed, Figure S5: Spatial locations of roadkill for three most prevalent species, Figure S6: Predicted roadkill rate of Bennett’s wallaby from GAM versus Section, Figure S7: Predicted roadkill rate of Bennett’s wallaby from GAM versus Period midpoint, Table S1: Roadkill numbers by all species and period.
