# Supplementary material for: A Trial of a Solar-Powered, Cooperative Sensor/Actuator, Opto-Acoustical, Virtual Road-Fence to Mitigate Roadkill in Tasmania, Australia
Source: Animals (Basel). 2019 Sep 30;9(10):752. doi: 10.3390/ani9100752 (PMC6827006; doi:10.3390/ani9100752)
Supplement: Supplementary file 1 [file animals-09-00752-s001.pdf]

Supplementary Material

1. VF operation
2. Monitoring regime
3. Data manipulation details
4. Traffic data
5. Raw spatial locations of three most prevalent roadkill species
6. Roadkill numbers by all species and period
7. GAM fitted cubic smoothing splines

1. VF operation

There are concerns about the theory of how the VF operates. The manufacturers state: ‘The acoustic sound of the warning sequence raises the attention of the animals and the flashing lights makes (sic) the animals feel uncomfortable and leave the road area’. Pavlovian (classical) conditioning requires that the unit’s alerting signal of the 4 kHz sound, a conditioned stimulus, would have to precede the flashing lights that produce the conditioned response of feeling uncomfortable, i.e., an aversive stimulus. In reality, sound travels at a slower rate, approximately 343 $m.s^{-1}$, than light, 300,000,000 $m.s^{-1}$. For animals positioned 30 m from the units, there is a delay of approximately 0.1 s. Thus, the sound arrives at the animal after the flashing lights, the opposite of what is required for the manufacturer’s explanation to have credence. Habituation, a form of non-associative learning in which an innate (non-reinforced) response to a stimulus decreases after repeated or prolonged presentations of that stimulus [1] could also be of concern, but could only be measured if the VF actually produces an innate response. Since in our study the VF produced no apparent effect on roadkill, i.e., the VF did not produce an innate response to make animals leave the roadway, habituation is not discussed as a reason for this effect.

Another concern is the loudness of the VF signal. Coulson and Bender mention that the sound frequency of the VF units is suitable but the intensity is unknown [43, in main text] . We used a Decibel X Pro-Sound Meter dBA (SkyPaw Co. Ltd, Hanoi Vietnam, Mobile app) noise detector, with unlocked frequency weighting A, to record the loudness (intensity) of the auditory output signal from the VF, the loudness of road traffic and the loudness of cicadas *(Cicadoidea)* and frogs *(Anura)* signalling at a distance of 12.5 m from the VF units and on the road verge between two VF units. The measurements of the road traffic and the cicadas and frogs were taken during the period from dusk into night-time at the trial site and measurement of the virtual fence unit in a secluded indoor area with low background noise, of approximately 32 dBA, and measured at a distance of 12.5 m from the unit.

Figure S1. Measurement of sound volume for general traffic, general background, and virtual fence.

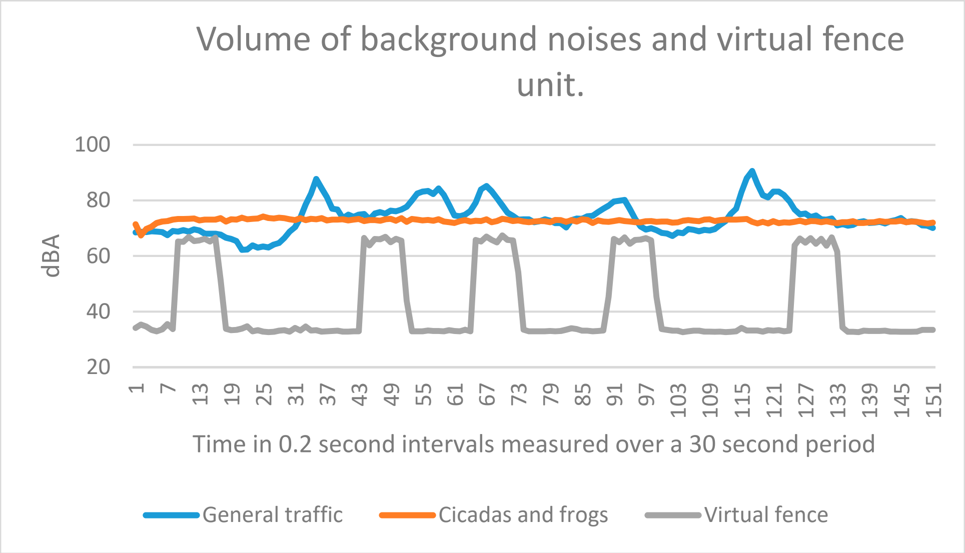

As illustrated, the peak output volume of the VF unit (68dBA), is approximately 5dBA below the noise of cicadas, frogs, and general background noise (73dBA). This is well below the sound volume of vehicles (91dBA) shown as peaks in the graph in Figure S1. It may well be that competing background noises do not allow the animals to hear or discriminate the 4 or 8 kHz audio signal of the VF units. Human hearing is most sensitive in the 2–5 kHz range and the Pro Sound measuring instrument was weighted for this. Although there is a paucity of auditory research on Tasmanian native animals, the hearing sensitivity of kangaroos and wallabies is in a similar range, 2 kHz–3.5 kHz, to that of humans [2]. Thus, discrimination would be extremely difficult when the background noise level is higher than that of the VF units. In correspondence with the current research team, the manufacturers stated: *'VF systems work most effective at low to medium dense traffic. If the traffic becomes nearly uninterrupted, the system cannot guide animals safely'*.

A further reason why the VF may be ineffective is that animals may ignore the auditory and visual stimuli of the VF units if there is an attractive resource that is more salient to them than the aversive LED flashing lights proposed by the manufacturers. This was illustrated to the researcher when carrying out night-time checks on the serviceability of the VF. A possum was seen feeding and to be ignoring the sound and flashing LED's of the VF unit (Figure S2).

Figure S2. Possum feeding at the base of a VF post, whilst the unit is operating.

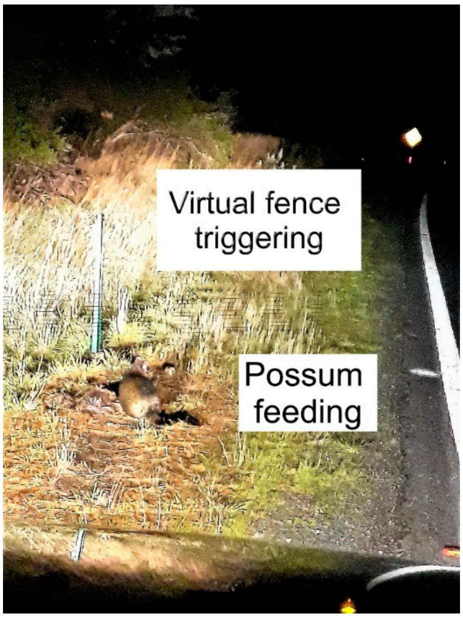

Similarly, on four separate occasions whilst checking the operation of the VF at night two Tasmanian pademelons, a Bennett's wallaby and a common brush-tailed possum were seen to be stationary on the roadside verge ignoring the sound and light emitted by the VF whilst it was triggering. It is recommended that further research investigates the efficacy of an increase in the volume of the auditory output of the VF units. Additionally, there seems to be merit in replacing the current 4 kHz and 8 kHz tones with a biologically significant auditory signal and adding a delay so that it follows the LED flashing lights by one to two s. A similar method was trialled in Poland to reduce the risk of train collisions with animals [3]. It was observed that 85%–93% of wild mammals escaped an approaching train. Biedenweg et al. [4] also reported that utilising both biologically significant sounds, such as a kangaroo alarm foot thump and Australian Raven (*Corvus coronoides*) call, and artificial sounds such as a bull whip crack, solicited an aversive reaction in western grey kangaroos (*Macropus fulliginosus*).

Operating the VF units in the trial

Switching is managed by the use of a paperclip inserted into a small entrance hole on the underside of the unit. There are three modes that can be engaged: Off, a 4 Khz so-called rural tone and an 8 Khz urban tone. Identification of the mode is by a series of different auditory beeps emitted by the unit. After switching off all the units, a subsequent check of the units the next day revealed that three were in the incorrect mode and so were only then switched to the off position. Discriminating the three modes is difficult when there is heavy traffic because the sound of the VF is compromised by the loudness of the vehicles.

The method of checking recommended by the manufacturers was to attend in daylight and place a black bag over the unit, to wait two min and then expose the unit to natural light to trigger the auditory and visual signals. This proved unreliable and time-consuming. The method eventually adopted was to undertake the checking at night with the use of vehicle headlights on the researcher's car. The car was driven onto the verge and stopped. After a ten s pause, with the headlights switched off, and with the car windows down, the units were triggered by a flash of the headlights enabling the sound and light emitted by the units to be checked for efficacy.

2. Monitoring regime

A car was furnished with a flashing amber safety lamp on its roof and a Caution, Frequently Stopping Vehicle sign on its rear. The researchers wore Australian Standard 1742.3 devices, i.e., Class D – Outdoor daytime use only, fluorescent high-visibility material vests.

There is no verge at the buffer zone sites, so the researchers walked the 4 km of these sections always facing the oncoming traffic and keeping 3 m from the roadway. For the rest of the study zone, the car was driven off the road along the wide verge at low speed which enabled roadkill to be spotted. When roadkill was observed, the car was stopped and used to protect the researcher as they left the vehicle to record a photograph, GPS, time, and date. The sightlines on the roadway provided a minimum of 10 s to see approaching vehicles with the speed of vehicles at the maximum speed limit of 100 km/$^{h-1}$. The work was classed as of short duration so was undertaken without delineation (e.g., warning signs, advisory speed signs, or line marking). The vehicle was positioned so that drivers travelling in either direction could see its flashing amber light as an extra safety precaution when the researcher was out of the vehicle.

In the unlikely, but possible, event of an animal roadkill victim being seen on the roadway that appeared to have live pouch young (joey), the victim was dragged to the verge at least 3 m from the roadway for examination. This procedure to remove the roadkill animal took no more than five to eight s, so a 20 s gap in the traffic was used for this purpose. Under the regulations, Standards Australia AS 1742.3-2009 Manual of uniform traffic control devices Traffic control for works on roads, '*a lookout person may be dispensed with if the work will not take more than ten seconds and approaching traffic can be seen for a distance away equal to 20 seconds travel time*' [5] (Pt 3).

3. Data manipulation details

Standardisation of rates was carried out as either a "month" standardisation given by 30*raw count/{(number of days in period)*(section length, km)} giving roadkill rate number.month^{-1}.km^{-1}, or as a "traffic count" standardisation given by 10^5*raw count/{(traffic count in period)*(section length, km)} giving roadkill per 100,000 trips.km^{-1} where the traffic count was all vehicles travelling between the hours of dusk and dawn. Times of sunset and sunrise for each day between 25 March and 20 August, used to calculate daily traffic counts between dusk and dawn (i.e., taken as the time of sunset and sunrise, respectively), were obtained using the Geoscience Australia National Mapping Division's sunrisenset program (version 2.2) for latitude -42°58'00" and longitude 147°14'00" and adjusted from Australian Eastern Standard Time to Australian Eastern Daylight-saving Time where necessary [6].

4. Traffic data

Figure S3. Daily traffic counts between dusk and dawn showing eastbound and westbound 26 March until 20 August 2018.

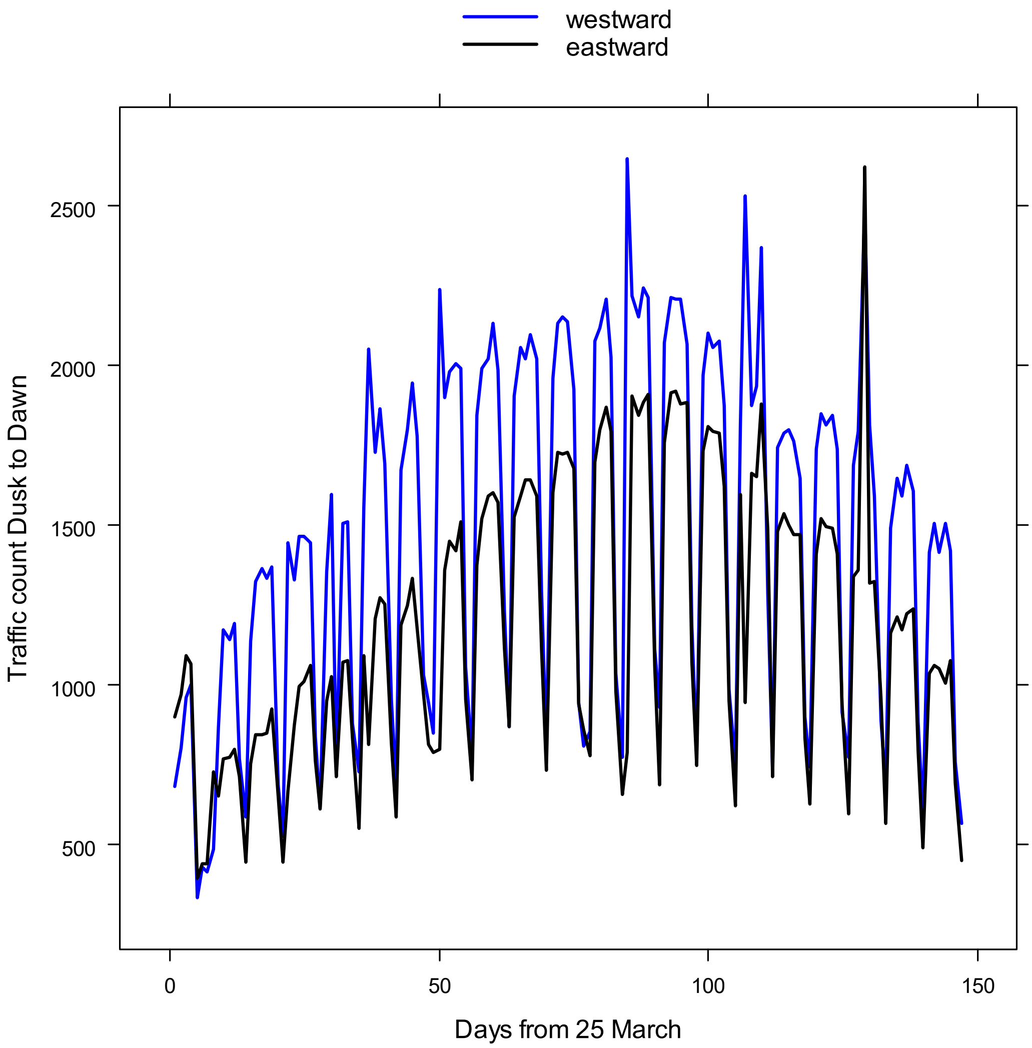

Figure S4. Average and maximum daily traffic speed between dusk and dawn by direction from the start of the trial. The average daily speed (thick black line) plus or minus one standard deviation (calculated daily) (thin blue lines) and daily maximum speed (thick red line). The thin vertical dashed lines represent the start date for each of the six periods shown in Table 1. The strong dip in speeds starting at Day 47 and lasting for three to four days corresponds to the extreme rainfall event (twice the previous record for a single day) that occurred for Hobart and surrounds starting on the evening of 10 May and resulted in erosion and large amounts of gravel washed on to the highway.

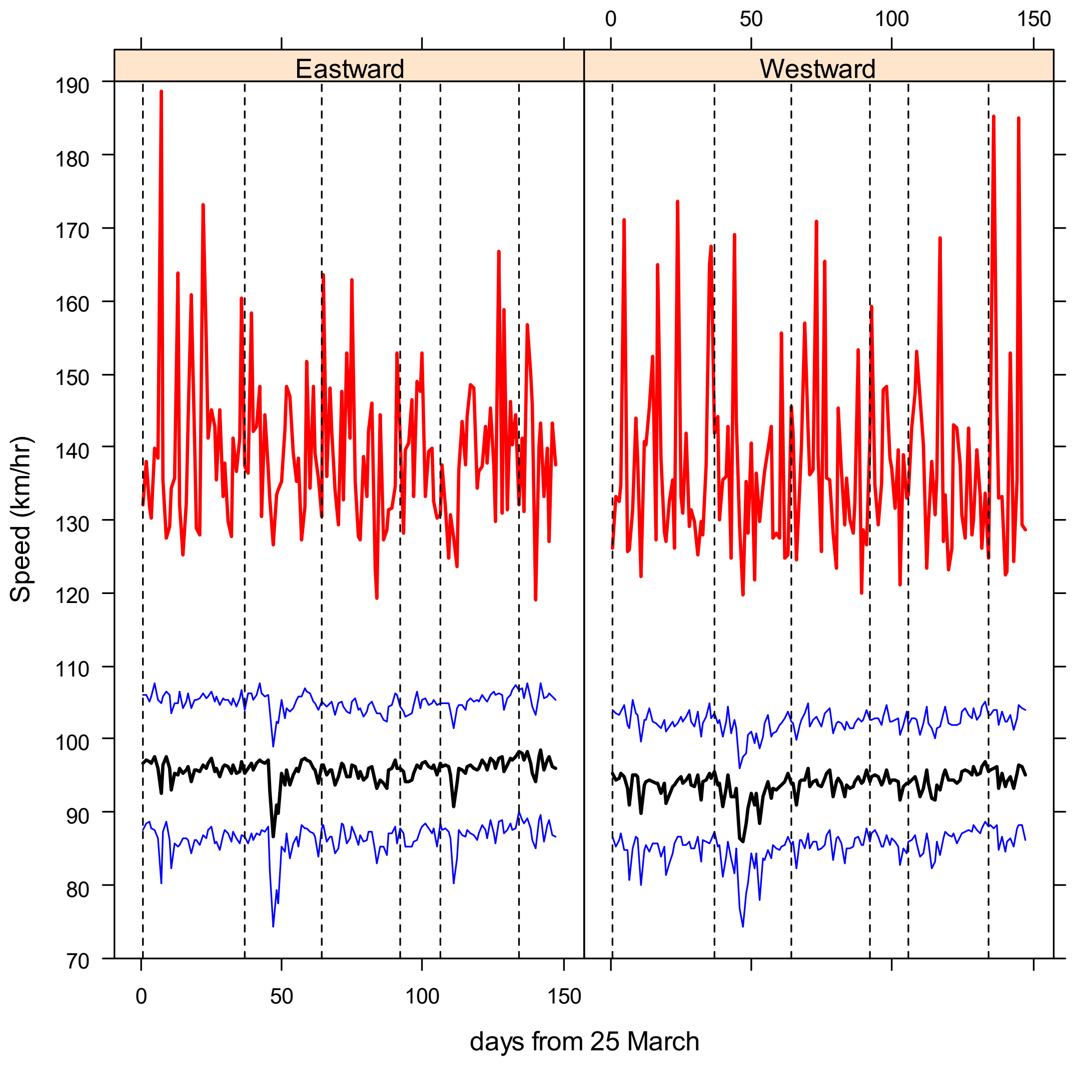

5. Raw spatial locations of three most prevalent roadkill species

FigureS 5. Spatial locations of roadkill for three most prevalent species of Bennett's wallabies (BW) *(Notamacropus rufogriseus)*, Tasmanian pademelons (TP) *(Thylogale billardierii)* and common brush-tail possums (BP) *(Trichosurus vulpecula)* .

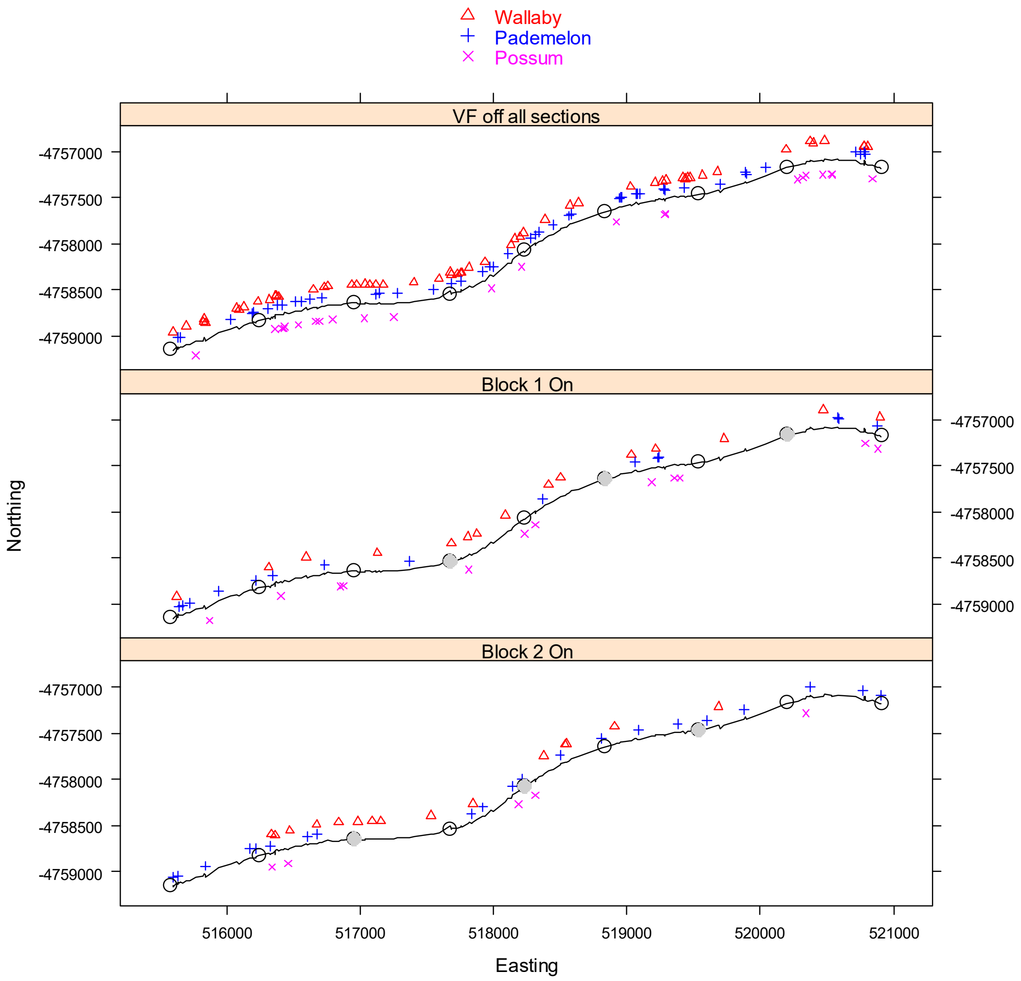

Panels show the locations (Northing, i.e., latitude and Easting, i.e., longitude) for Periods 1, 2, 4, and 6 (total of 70 days) when the VF was switched off on Sections 2 to 7 and for each of Period 3 (Block 1 sections On) and Period 5 (Block 2 sections On) with each a period of 28 days. Sections 1 to 8 are delineated by the circles with the start of sections (proceeding westward) when switched on shown as filled circles. The Northing of roadkill locations have been offset slightly for clarity. The right-hand side of the graph starts at the eastern buffer Section 1.

6. Roadkill numbers by all species and period

Table S1. Roadkill numbers by all species and period with On-Off Periods 3 and 5 disaggregated to On and Off Blocks excluding buffer sections 1 and 8 (Block 1: Sections 2, 4, 6; Block 2: Sections 3, 5, 7). The periods when the VF was switched on are italicised.

**Period label**	**Pre-trial**	**Pre_All_Off**	**Block1_On**		**Post1_All_Off**	**Block2_On**		**Post2_All_Off**	
Period No	1	2	*3*		4	*5*		6	
Start Date 2018	26/03	1/05	*28/05*		25/06	*9/07*		6/08	
End Date 2018	8/04	28/05	*25/06*		9/07	*6/08*		20/08	
Period (days)	14	28	*28*		14	*28*		14	
VF on vs off			**Block2_Off**	**Block1_*On***		**Block1_Off**	**Block2_*On***		
**Species**									**Species Total**
Bennett's wallaby (*M.rufogriseus*)	16	18	8	4	6	9	6	1	68
rufous-bellied pademelon (*T.billardierii*)	6	13	6	3	7	6	7	10	58
common brush-tailed possum (*T.vulpecula*)	7	4	7	2	1	1	3	3	28
bettong (*Bettongia aimardi*)	0	2	0	0	0	0	2	1	5
bandicoot (*Perameles gunnii*)	0	1	0	1	0	2	0	0	4
kookaburra (Dacelo)	2	1	0	0	1	0	0	0	4

Tasmanian native_hen (*Tribonyx mortierii*)	0	1	0	0	1	0	0	1	3
ringtail possum (*Pseudocheirus peregrinus*)	0	1	0	1	0	0	0	0	2
Tasmanian devil (*S. harrisii*)	0	1	0	0	0	0	1	0	2
Period Total	31	42	21	11	16	18	19	16	174

7. GAM fitted cubic smoothing splines

Figure S6. Predicted roadkill rate of Bennett's wallaby from GAM versus Section for VF_on_vs_off set to "Off" and Period_mid set to 100. Single SE bounds shown as dashed lines.

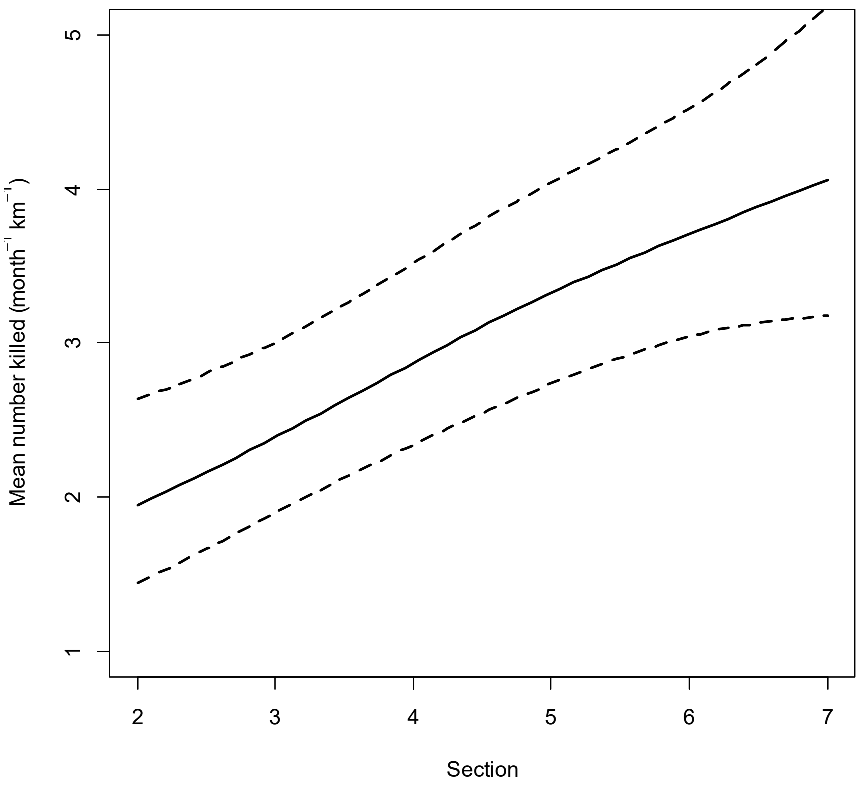

Figure S7. Predicted roadkill rate of Bennett's wallaby from GAM versus Period midpoint for VF_on_vs_off set to "Off" and Section set to 2. Single SE bounds shown as dashed lines.

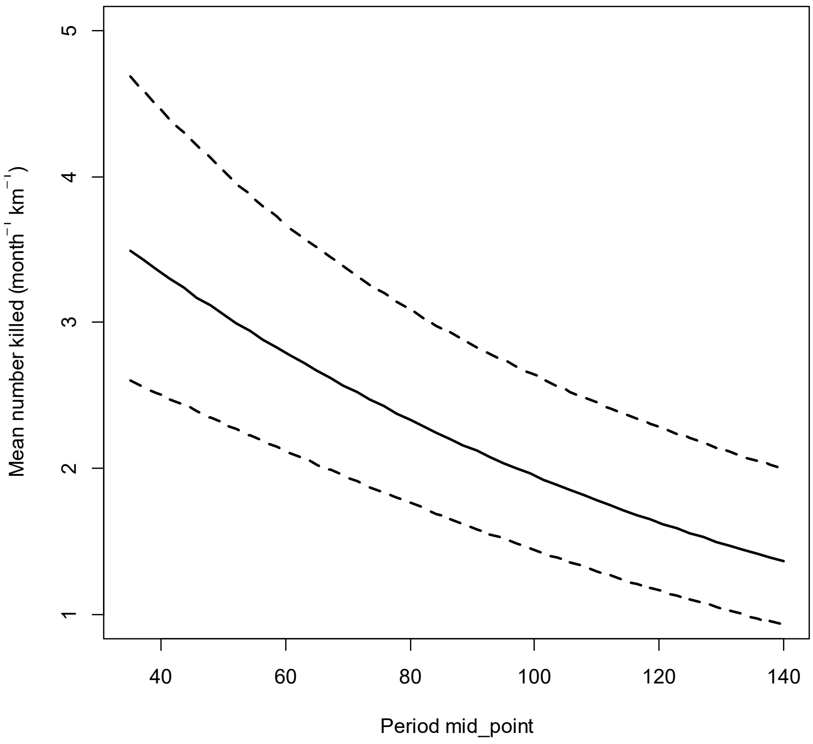

References

1. Bouton, M.E. *Learning and Behavior : A Contemporary Synthesis*, 2nd ed.; Sinauer Associates, Inc.: Sunderland, MA, USA, 2016.
2. Bender, H. *Auditory Stimuli as a Method to Deter Kangaroos in Agricultural and Road Environments*; Department of Zoology, University of Melbourne: Melbourne, Australia, 2005.
3. Babińska-Werka, J.; Krauze-Gryz, D.; Wasilewski, M.; Jasińska, K. Effectiveness of an acoustic wildlife warning device using natural calls to reduce the risk of train collisions with animals. *Transp. Res. Part D* **2015**, *38*, 6–14.
4. Biedenweg, T.A.; Parsons, M.H.; Fleming, P.A.; Blumstein, D.T.; Somers, M. Sounds scary? Lack of habituation following the presentation of novel sounds. *PLoS ONE* **2011**, *6*, e14549.
5. Standards Australia. AS 1742.3-2009 Manual of uniform traffic control devices traffic control for works on roads. Availabe online: https://infostore.saiglobal.com/en-au/Standards/AS-1742-3-2009-126126_SAIG_AS_AS_275158/ (accessed on 12 February 2019).
6. Geoscience Australia. Compute sunrise, sunset & twilight times. Available online: http://www.ga.gov.au/bin/geodesy/run/sunrisenset (accessed on 11 March 2019).
